# Building a sense of community in service-learning and its association with college students’ prosocial behavior and social responsibility

**DOI:** 10.3389/fpsyg.2026.1796279

**Published:** 2026-03-24

**Authors:** Wei Xiao

**Affiliations:** College of Veterinary Medicine, Yangzhou University, Yangzhou, Jiangsu, China

**Keywords:** educational significance, mediation analysis, prosocial behavior, sense of community, service-learning quality, social responsibility

## Abstract

**Introduction:**

Although service-learning is widely used in higher education, the psychological mechanisms through which it is associated with students’ social development remain unclear. The present research aims to examine whether a sense of community mediates the relationship between the quality of service-learning and students’ prosocial behavior and social responsibility.

**Methods:**

A cross-sectional questionnaire survey was conducted with 735 Chinese university students who had participated in service-learning. Structural equation modeling and the Bootstrap method were utilized to test the mediation effects, and independent samples t-tests were used to analyze the impact of team size.

**Results:**

The results showed that: (1) the quality of service-learning was significantly and positively correlated with a sense of community (*r = 0.582, p < 0.001*); (2) sense of community partially mediated the association between service-learning quality and social responsibility, with the mediation effect accounting for 47.8% of the total effect (indirect effect = 0.244, 95% CI [0.189, 0.302]); (3) the mediation effect of sense of community on the association with prosocial behavior accounted for 62.1% of the total effect; and (4) team size had a significant impact on the sense of community, with small teams (2–10 members) scoring significantly higher than large teams (*t = 7.93, p < 0.001*).

**Discussion:**

The present research provides a preliminary exploration of the role of sense of community in service-learning, finding that it functions as a key mechanism linking the external educational environment to the student’s internal value system. The findings suggest that by building an emotional community, educators can help students rediscover engagement and meaning in learning, thereby facilitating a shift from passive participation to active commitment.

## 1 Introduction

### 1.1 Problem statement

A growing sense of “academic alienation” is pervading higher education globally, where students increasingly perceive a disconnect between classroom learning and real-life application. Under a highly competitive and standardized assessment system, many students feel that classroom learning is disconnected from real life. Learning is often alienated into a mere means of obtaining credits or degrees, leading to a loss of intrinsic joy and motivation. Recent studies indicate that this sense of alienation is closely related to increasingly severe mental health problems (such as anxiety and depression), urgently requiring educational interventions to rebuild students’ sense of connection (Anderson et al., 2025). How to help students rediscover curiosity, engagement, and meaning in the educational process has become a pressing frontier issue in educational psychology (Ma et al., 2025).

Service-Learning, which integrates community service with academic curricula, offers a unique pedagogical approach in this context. Its value lies in breaking down campus walls, requiring students to apply classroom knowledge to meet real community needs. This is not only an extension of knowledge but also a process for students to find their place in a larger social system. The latest meta-analytic studies confirm that service-learning can effectively enhance students’ civic awareness, social responsibility, and critical thinking skills, with this effect being universal across different cultural backgrounds (Guanlao et al., 2025; Compare and Albanesi, 2023).

However, the effectiveness of service-learning in practice varies significantly. Under the same activity design, some students experience the “joy of helping others” and a “sense of being needed” in service, showing stronger social responsibility; while others have a bland experience or even feel burnt out. What are the psychological mechanisms behind this difference? Why does the same external context produce vastly different experiences of meaning in different individuals? While numerous studies have documented the positive outcomes of service-learning, the psychological pathways explaining how these outcomes are achieved remain underexplored. This has led to an insufficient understanding of “why it works”.

The present research posits that service-learning, by applying academic knowledge to solve real social problems, provides a field for “meaning-making” for students. In this field, mere participation is not enough; the key is whether students perceive a supportive social environment. To address this gap, this study introduces the concept of Sense of Community (SOC), a core construct in community psychology, as a potential psychological bridge. McMillan and Chavis define sense of community as an individual’s sense of belonging to their group. From a psychosocial perspective, a sense of community can be seen as a supportive force. When students feel “I belong here” and “our actions are meaningful” within their team, this secure emotional connection can effectively reduce learning anxiety, allowing students to regain their passion and curiosity for society, and subsequently, spontaneously generate prosocial behavior and a sense of responsibility (Russo et al., 2025; Marino et al., 2025). This study uses the “joy of learning” not as a formal theory, but as a conceptual framework to emphasize the importance of positive affective experiences in education, which aligns with established theories such as Self-Determination Theory’s focus on relatedness.

Existing research has focused more on the “moderating role” of sense of community, neglecting its “mediating role” as an emotional engine. In other words, how does a high-quality service experience become associated with students’ internal life meaning and social responsibility through the emotional medium of “sense of community”? To address this underexplored area, the present study constructs and tests a mediation model. We construct a statistical mediation model of “service-learning quality → sense of community → social responsibility/prosocial behavior,” using a large-sample questionnaire survey and structural equation modeling to systematically test the mediating role of sense of community. This is not only a test of a mechanism but also an attempt to reveal how the educational environment can awaken students’ intrinsic engagement, thereby providing empirical evidence for “rediscovering meaning in education”.

### 1.2 Significance of the study

#### 1.2.1 Theoretical significance

Shifting from an “input–output” to a “meaning-making” Model. For a long time, service-learning research has mainly focused on the linear relationship between activity participation (input) and learning outcomes (output). By introducing the psychological variable of sense of community, the present research explores the psychological process of how service-learning is associated with a shift from an “external task” to an “internal identity” for students, enriching the explanation of the sources of “engagement” and “meaning” in education.

Deepening the application of sense of community theory in educational contexts. It investigates how, in short-term practical teams, a sense of community acts as a “secure base,” potentially stimulating the individual’s motivation to explore the external world (prosocial behavior), and provides empirical evidence for the cross-contextual application of this theory.

Providing a new perspective for understanding the social development of university students. It suggests that “social responsibility” is not merely a product of preaching but a psychological quality that naturally emerges from joyful social interaction (positive learning experiences) and a sense of belonging. This implies that the essence of the socialization process is the establishment of relationships and emotional resonance.

#### 1.2.2 Practical significance

Providing a humanistic basis for optimizing service-learning design. The research findings will guide educators on how to enhance educational outcomes by creating a warm climate in teams, making the educational process itself full of care and warmth, and thus allocating resources more scientifically.

Improving the evaluation system for practical education. The present research shows that psychological indicators such as a sense of community are important predictors of educational effectiveness. Incorporating them into the evaluation system is also a reflection of focusing on students’ psychological experiences and respecting their subjectivity.

Providing a pathway for building the “affinity” of ideological and political education. In the context of Chinese higher education, the present research reveals how to eliminate the “sense of alienation” in education by building an emotional community, achieving a shift from “being told what to do” to “being willing to do it.” This provides a psychological perspective and reference for enhancing the attractiveness and appeal of ideological and political education and for building a more compassionate “big ideological and political course”.

### 1.3 Research objectives and hypotheses

Based on the analysis above, the present research sets the following objectives:

Research objective 1: to examine the impact of service-learning quality on university students’ social responsibility and prosocial behavior.
Research objective 2: to verify the mediating role of sense of community in the above process (i.e., the psychological transformation mechanism).
Research objective 3: to explore the influence of contextual factors such as team size and service recipients on the sense of community.

Accordingly, the following research hypotheses are proposed:

*H1:* Service-learning quality has a significant positive association with sense of community.

*H2:* Sense of community has a significant positive association with prosocial behavior.

*H3:* Sense of community has a significant positive association with social responsibility.

*H4:* Sense of community plays a mediating role in the association between service-learning quality and social responsibility.

*H5:* Sense of community plays a mediating role in the association between service-learning quality and prosocial behavior.

*H6:* Team size has a significant impact on sense of community, with small teams having a higher sense of community than large teams.

## 2 Literature review

### 2.1 Service-learning: from volunteer service to meaning-making

Service-learning originated in the United States in the 1960s. The effectiveness of service-learning stems from its three integrated components, where meaningful service activities are explicitly linked to the curriculum and deepened through structured reflection. It is precisely these three integrated components that elevate the activity from simple volunteering to a platform for “holistic education.” From a psychosocial perspective service-learning allows students to become active interactors with the real world, rather than passive recipients of knowledge. Service-learning allows students to become active interactors with the real world, rather than passive recipients of knowledge. Bringle and Hatcher emphasized that this interaction not only deepens academic understanding but is also an important source for students to gain a sense of self-efficacy in a social context and a sense of meaning. When students discover that their knowledge can solve practical problems, dry theories are transformed into vivid practice, and their intrinsic motivation for learning can be stimulated (Gutzweiler et al., 2022).

In China, with the deepening of the concepts of “fostering virtue through education” and “three-all education,” service-learning is gradually transforming from a traditional auxiliary means of ideological and political education into a psychological intervention model for reshaping students’ values and social responsibility. High-quality service-learning, by providing clear goals and opportunities for deep reflection, helps students understand the relationship between the “small self” and the “greater self” in action, thus transcending mere volunteer service to become a profound educational life experience (Zhen, 2025; Yan et al., 2025).

### 2.2 Sense of community: the “social cornerstone” of the positive learning experiences

Sense of Community is a core concept in community psychology. McMillan and Chavis’s foundational theory conceptualizes sense of community through four dimensions. For the present research, the concepts of “membership” and “shared emotional connection” are particularly relevant, as they form the “secure base” for students understanding of group dynamics. In line with Self-Determination Theory (SDT), which posits relatedness as a core psychological need for fostering intrinsic motivation, the present research particularly focuses on the psychological function of sense of community as a “secure base” (Li et al., 2025).

Membership and Belonging: When university students feel accepted (Membership) in a service team, this sense of belonging significantly reduces the anxiety of facing an unfamiliar social environment, providing a psychological safety net for “positive engagement.” This psychological support is particularly crucial for Chinese university students who face the dual pressures of employment and academics (Ma et al., 2025).

Emotional Connection and Meaning: In the educational context, “joy” often originates from deep interpersonal resonance. The Emotional Connection within a sense of community prevents students from being solitary actors. When students perceive an emotional bond with their peers and service recipients, the act of “serving others” is elevated from a tedious external obligation to a Meaningful Experience filled with a sense of accomplishment (Portes, 2024). This positive emotion, supported by the community, is the source of learning Engagement (Liao et al., 2023).

Therefore, in the collaborative context of service-learning teams, a sense of community is not only a manifestation of organizational cohesion but also a key psychological variable that is associated with students’ shift from “passive participation” to “active exploration” (Shea and Bidjerano, 2010). While sense of community is a multidimensional construct, the current study focuses on the “shared emotional connection” dimension, conceptualizing it as the core of a positive interpersonal climate that underpins this shift. This focus aligns with the Community of Inquiry framework, where social presence is seen as a prerequisite for the self-regulation and self-efficacy necessary for deep engagement.

### 2.3 Prosocial behavior and social responsibility: the outcomes of meaning-making

Prosocial behavior and social responsibility are often considered output variables of service-learning. However, in the present research, they are seen as the natural expression of students finding “self-meaning” through community interaction.

Prosocial behavior as an “overflow of joy”: research shows that when individuals are in a positive emotional community, they are more likely to experience the “warm-glow effect,” where the act of helping itself brings pleasure. This behavior, based on reciprocity and empathy, is the overt action that follows the student’s experience of the “joy of helping” (Alfirević et al., 2023). Recent studies on Chinese and other Asian students have also found that this “helping” experience can effectively buffer the negative emotions brought by academic stress and enhance subjective well-being (Kor et al., 2025).

Social responsibility as the “internalization of values”: Social responsibility is a relatively stable attitudinal tendency. However, theories of adult development and experiential learning suggest that such attitudes are not immutable and can be shaped by significant life events, including impactful educational experiences like service-learning (Arnett, 2000). In an environment with a strong sense of community, the public values of the team are more easily internalized by individuals as personal qualities through observational learning and social identity mechanisms (Russo et al., 2025). This is not just a cognitive understanding of social obligations but a higher-order Search for Meaning—where individuals confirm their value to society through service (Marino et al., 2025).

### 2.4 Derivation of theoretical hypotheses: the affective mediation pathway

Existing research has mostly focused on the direct effects of service-learning, i.e., “participation leads to effectiveness.” However, we believe this view ignores the internal psychological transformation mechanism. This is like a “black box”: how does a high-quality service design (input) relate to students’ social growth (output)?

The present research proposes that a sense of community plays the role of an “emotional converter” in this process. Based on frameworks such as Kolb’s (2014) experiential learning theory and McMillan and Chavis’s (1986) theory of sense of community, we hypothesize a specific sequence: a well-structured educational environment (high-quality service-learning) fosters a positive interpersonal climate (sense of community), which in turn is associated with the development of positive social attitudes and behaviors.

A high-quality service-learning environment (meaningful service, reflection, curriculum integration) provides a highly structured interactive platform, which is expected to promote trust and dependence among members (H1: Service-learning quality → Sense of community).

This positive community experience satisfies individuals’ needs for belonging and emotion, making students more willing to give back to society (prosocial behavior) and take on responsibility (social responsibility), thus achieving a link from “environmental support” to “personal qualities” (Nichol et al., 2024) (H4, H5: Mediating role of sense of community). While alternative models, such as sense of community acting as a predictor of perceived service quality, could be conceivable, the current study’s framework is grounded in the pedagogical principle that the designed environment precedes the participant’s subjective experience within it, a perspective aligned with the Stimulus-Organism-Response (S-O-R) framework in environmental psychology (Mehrabian and Russell, 1974).

### 2.5 Reframing the research question

In summary, although existing research has confirmed the benefits of service-learning, it has the following shortcomings:

Emphasis on cognition over emotion: It overlooks the core role of a “pleasant community experience” in value guidance.
Lack of process mechanisms: It fails to reveal the “psychological bridge” connecting the external environment with internal growth.

Therefore, the present research aims to explore how to enhance the educational effectiveness of service-learning by improving the sense of community through the construction of a statistical model: “Environmental Evaluation (Quality) → Affective Mediation (Sense of Community) → Meaningful Output (prosocial/responsibility).” This will help answer the fundamental question of “how to make the educational process not only effective but also full of meaning and joy.” This not only helps to deepen the understanding of the mechanisms of practical education but also provides empirical evidence for promoting more humanistic teaching models in global higher education.

## 3 Research design and methods

### 3.1 Participants

The present research targeted university students who had participated in service-learning. A combination of convenience sampling and snowball sampling was used to distribute electronic questionnaires through class groups, club groups, and other channels across several universities in eastern China. The survey was conducted from October to November 2025.

The questionnaire included a screening question, allowing only students who had “participated in service-learning in the past year” to proceed. A total of 760 questionnaires were collected. After data cleaning, 25 invalid questionnaires (e.g., excessively short response times, patterned responses, failure to meet screening criteria) were removed, resulting in 735 valid questionnaires, for a validity rate of 96.7%. The basic characteristics of the sample are shown in Table 1.

**Table 1:** Basic information of the sample (*N* = 735).

Category	Option	Number	Percentage
Gender	Male	302	41.1%
	Female	433	58.9%
Year	Freshman	251	34.1%
	Sophomore	264	35.9%
	Junior	149	20.3%
	Senior	56	7.6%
	Graduate student	15	2.0%
Major	Humanities & social sciences	294	40.0%
	Science, engineering, agriculture & medicine	301	41.0%
	Arts & sports	72	9.8%
	Other	68	9.2%
Service duration	Within 1 day	223	30.3%
	2–7 days	291	39.6%
	8–30 days	152	20.7%
	More than 1 month	69	9.4%
Team size	2–5 members	187	25.4%
	6–10 members	254	34.6%
	11–20 members	181	24.6%
	More than 20 members	113	15.4%

As shown in the table, the sample is relatively balanced in terms of gender, year of study, and major. Underclassmen (freshmen and sophomores) account for about 70%, which is consistent with the reality that practical activities in universities are mainly aimed at lower-year students. The service duration was predominantly short-term projects (2–7 days), and the team sizes were mostly small to medium (6–10 members).

### 3.2 Research instruments

The present research used a questionnaire survey for data collection. The selection of scales was based on classic instruments in the field of higher education service-learning and the latest systematic reviews (Mahmud and Ismail, 2024). The questionnaire consisted of five parts: demographic and project characteristic information, Service-Learning Quality Scale, Sense of Community Scale, Prosocial Behavior Scale, and Social Responsibility Scale. Except for the first part on basic information, all scale items used a Likert 5-point scale (1 = “Strongly Disagree” to 5 = “Strongly Agree”). The complete survey questionnaire can be found in Appendix A.

#### 3.2.1 Service-learning quality

Adapted from the Service-Learning Scale by Bringle and Hatcher (1995) and drawing on Astin’s (1997) theoretical framework of student involvement. It includes 3 dimensions: “meaningful service,” “curriculum integration,” and “structured reflection,” with a total of 6 items.

#### 3.2.2 Sense of Community

Based on McMillan and Chavis’s (1986) four-dimensional theory of sense of community (membership, influence, integration and fulfillment of needs, and shared emotional connection), and revised with reference to the Brief Sense of Community Scale (BSCS) by Peterson et al. (2008) and the SCI Index Manual by Chavis et al. (2008), totaling 8 items.

#### 3.2.3 Prosocial Behavior

Primarily based on the Adult Prosocialness Scale by Caprara et al. (2005), with item design also incorporating the multi-level perspective on prosocial personality by Penner et al. (2014). It focuses on measuring two dimensions: “helping and sharing” and “empathy and caring,” with a total of 6 items.

Social Responsibility: Adapted from the classic Social Responsibility Scale by Berkowitz and Lutterman (1968), including two dimensions: “social obligation” and “civic engagement,” with a total of 6 items.

### 3.3 Data processing

SPSS 26.0 was used for data processing, including descriptive statistics, reliability and validity tests, correlation analysis, and difference testing. As team size and service type were identified as significant contextual factors, their influence was first examined through difference tests. The main analysis then employed the PROCESS macro (Model 4) with the bias-corrected percentile Bootstrap method was employed to test for mediation effects (Hayes, 2017), controlling for gender and grade. The significance level was set at 0.05.

## 4 Results and analysis

### 4.1 Data quality check

#### 4.1.1 Common method bias test

Given that the data for all variables were collected from the same source at a single point in time, common method bias (CMB) could be a potential concern. To address this, we employed both procedural controls and statistical tests.

Procedurally, we took steps to mitigate CMB during the survey design phase. The questionnaire assured participants of anonymity and the confidentiality of their responses. Additionally, the items for the independent, mediator, and dependent variables were placed in different sections of the questionnaire to create psychological separation.

Statistically, we first conducted Harman’s single-factor test as a preliminary check. An unrotated principal component analysis showed that the first factor explained 28.43% of the total variance, below the 40% threshold. While this provides initial reassurance, we further conducted a more robust full collinearity test, as recommended by Kock (2015). We generated variance inflation factors (VIFs) for all variables in our model. The results showed that all VIF values ranged from 1.52 to 2.11, which are well below the conservative threshold of 3.3. This indicates that multicollinearity is not an issue and further suggests that common method bias is unlikely to be a serious concern in this study. In combination, these procedural and statistical controls provide confidence that CMB does not pose a substantial threat to the validity of our findings.

#### 4.1.2 Reliability and validity test

Reliability analysis was conducted on each scale. As shown in Table 2, the Cronbach’s *α* coefficients for the four scales ranged from 0.764 to 0.818, indicating good internal consistency. To further assess the reliability and convergent validity of the measurement model, Composite Reliability (CR) and Average Variance Extracted (AVE) were calculated based on a Confirmatory Factor Analysis (CFA).

**Table 2:** Reliability and convergent validity results (*N* = 735).

Variable	Items	Cronbach’s α	CR	AVE
Service-learning quality (SLQ)	6	0.764	0.838	0.465
Sense of community (SOC)	8	0.791	0.875	0.472
Prosocial behavior (PSB)	6	0.818	0.882	0.556
Social responsibility (SR)	6	0.812	0.871	0.531

The Results in Table 2 show that all CR values ranged from 0.838 to 0.882, well above the recommended threshold of 0.70. Although the AVE values for Service-Learning Quality (0.465) and Sense of Community (0.472) were slightly below the 0.50 threshold, their CR values were significantly higher than 0.70. According to Fornell and Larcker (1981), if the CR is greater than 0.70, the convergent validity of the construct is still considered adequate even if the AVE is less than 0.50. Thus, the measurement scales demonstrated satisfactory convergent validity.

Furthermore, the CFA results demonstrated that the measurement model fitted the data well: χ^2^/df = 2.84, CFI = 0.926, TLI = 0.915, RMSEA = 0.051, and SRMR = 0.042. All item factor loadings were greater than 0.50 and statistically significant (*p < 0.001*), confirming the structural validity of the questionnaire.

Finally, discriminant validity was assessed using the Fornell-Larcker criterion. As shown in Table 3, the square root of the AVE for each construct (diagonal values) was greater than its correlation with any other construct, indicating that the four variables are distinct and have good discriminant validity.

**Table 3:** Discriminant validity results.

Variable	1	2	3	4
1. Service-learning quality	(0.682)			
2. Sense of community	0.582**	(0.687)		
3. Prosocial behavior	0.493**	0.615**	(0.746)	
4. Social responsibility	0.511**	0.598**	0.632**	(0.729)

Diagonal values in parentheses represent the square root of the AVE; ** *p* < 0.01.

### 4.2 Descriptive statistics

The means and standard deviations of each variable are shown in Table 4.

**Table 4:** Descriptive statistics of main variables (*N* = 735).

Variable	Mean	Standard deviation	Theoretical range
Service-learning quality	3.61	0.72	1–5
Sense of community	3.72	0.75	1–5
Prosocial behavior	4.02	0.68	1–5
Social responsibility	4.11	0.65	1–5

As can be seen from the table, the mean score for service-learning quality is 3.61, which is at a moderately high level. This suggests that students generally have a positive evaluation of the practical activities they participated in, but there is still room for improvement. The mean for sense of community is 3.72, slightly above the theoretical midpoint. The mean scores for prosocial behavior and social responsibility are both above 4.0, indicating that the university student population possesses a high level of moral character and civic awareness.

### 4.3 Difference test

#### 4.3.1 Differences in team size

Team size was divided into small teams (2–10 members, *N* = 441) and large teams (11 or more members, *N* = 294) for an independent samples t-test. The results are shown in Table 5.

**Table 5:** Differences in variables across different team sizes.

Variable	Small team (*N* = 441)	Large team (*N* = 294)	t-value	*p*-value
Service-learning quality	3.65 ± 0.71	3.55 ± 0.74	1.82	0.069
Sense of community	3.89 ± 0.68	3.47 ± 0.79	7.93	<0.001
Prosocial behavior	4.08 ± 0.65	3.93 ± 0.71	2.97	0.003
Social responsibility	4.16 ± 0.62	4.03 ± 0.68	2.71	0.007

**Figure 1:**
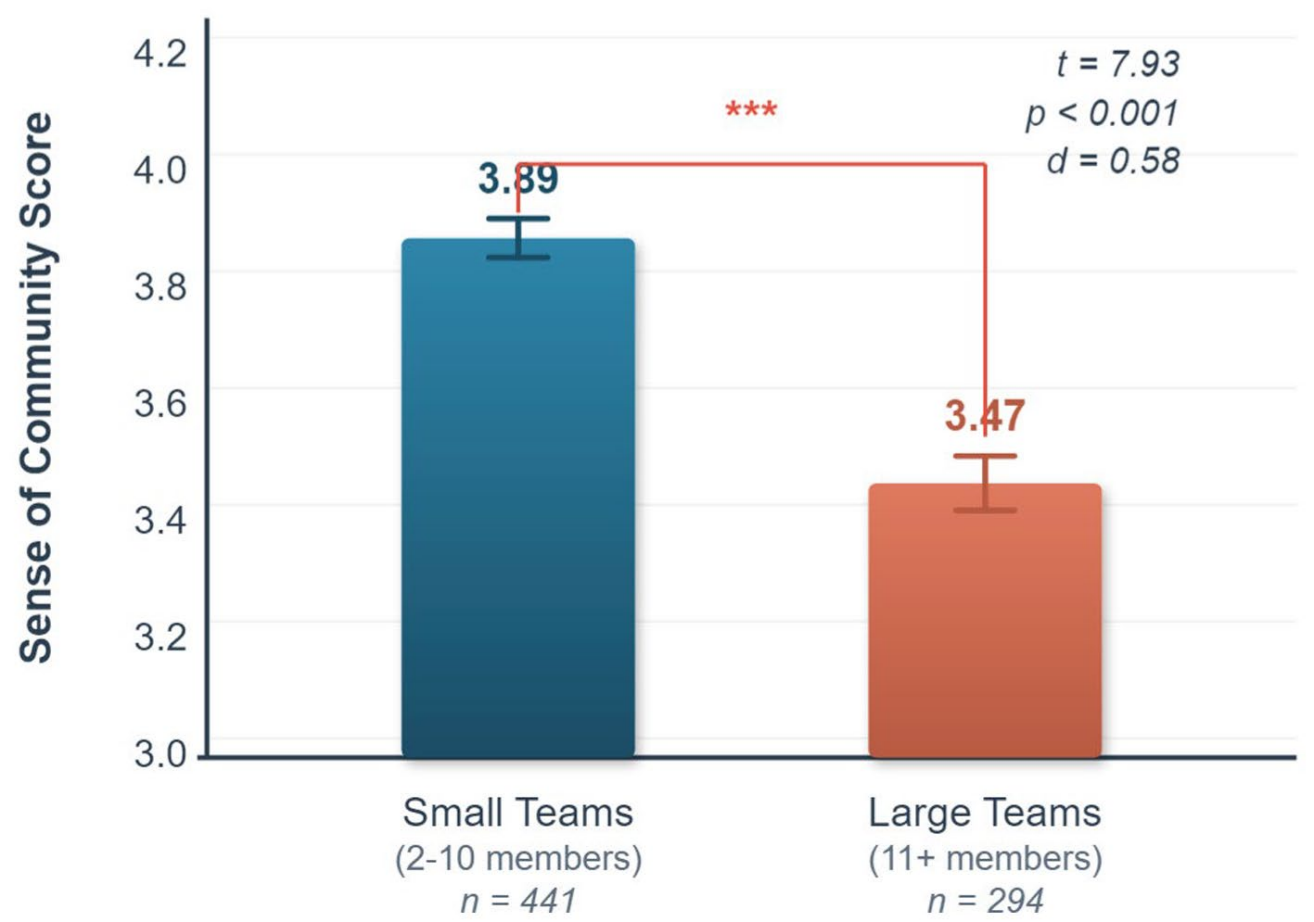
Comparison of sense of community across different team sizes.

The results show that small teams scored significantly higher than large teams on sense of community, prosocial behavior, and social responsibility. The difference in sense of community was the most significant (*t = 7.93*, *p < 0.001*). This validates hypothesis H6. Figure 1 visually displays this difference.

This finding lends empirical support to the theory of sense of community. In small teams, interactions among members are more frequent, making it easier to build trust and emotional connections. In large teams, the number of members is large, communication opportunities are fewer, and the “bystander effect” is more likely to occur, leading to a weaker sense of individual belonging. This aligns with the findings of Vázquez et al. (2025) on the importance of social skills and small-group interaction in enhancing learning competence.

#### 4.3.2 Differences in service recipients

Service recipients were divided into “person-centered” (children/elderly/disabled/community, *N* = 469) and “task-centered” (rural revitalization/environmental protection/other, *N* = 266) for comparison. The results are shown in Table 6.

**Table 6:** Differences in prosocial behavior across different service recipients.

Service recipient type	Sample size	Mean of prosocial behavior	Standard deviation	t-value	*p*-value
Person-centered	469	4.12	0.64	4.56	<0.001
Task-centered	266	3.85	0.71		

The results show that students participating in “person-centered” services had significantly higher scores in prosocial behavior than those in “task-centered” services. This may be because activities that directly serve people (such as teaching support, caring for the elderly) are more likely to stimulate empathy and a willingness to help. In fact, a systematic meta-analysis has already confirmed that service-learning has a significant positive impact on improving university students’ empathy (Gordon et al., 2022; Yan et al., 2025). In their interactions with service recipients, students can personally feel the needs of others and the value of their own actions. As Vine and Greenwood (2022) pointed out, this interaction of community solidarity creates “spaces of recognition” where students deeply understand the commonality that “we are all human beings,” thereby enhancing altruistic tendencies. This is consistent with the views of Reynolds et al. (2022) on the bidirectional promotion of mental health through the emotional flow between caregivers and care recipients.

#### 4.3.3 Differences in service duration

To examine the influence of service duration, we conducted an independent samples t-test comparing students in short-term (1–7 days, *N* = 514) and long-term (8 days or more, *N* = 221) service-learning projects. As shown in Table 7, students in long-term projects reported significantly higher scores in sense of community (*t = −4.29, p < 0.001*), prosocial behavior (*t = −2.57, p = 0.010*), and social responsibility (*t = −2.65, p = 0.008*). These findings suggest that sustained engagement in service activities is more conducive to fostering emotional connections within the team and internalizing social values.

**Table 7:** Differences in variables across different service durations (*N* = 735).

Variable	Short-term (*N* = 514)	Long-term (*N* = 221)	t-value	*p*-value
Sense of community	3.64 ± 0.77	3.90 ± 0.69	−4.29	<0.001
Prosocial behavior	3.98 ± 0.69	4.12 ± 0.64	−2.57	0.010
Social responsibility	4.07 ± 0.66	4.21 ± 0.62	−2.65	0.008

### 4.4 Correlation analysis

**Figure 2:**
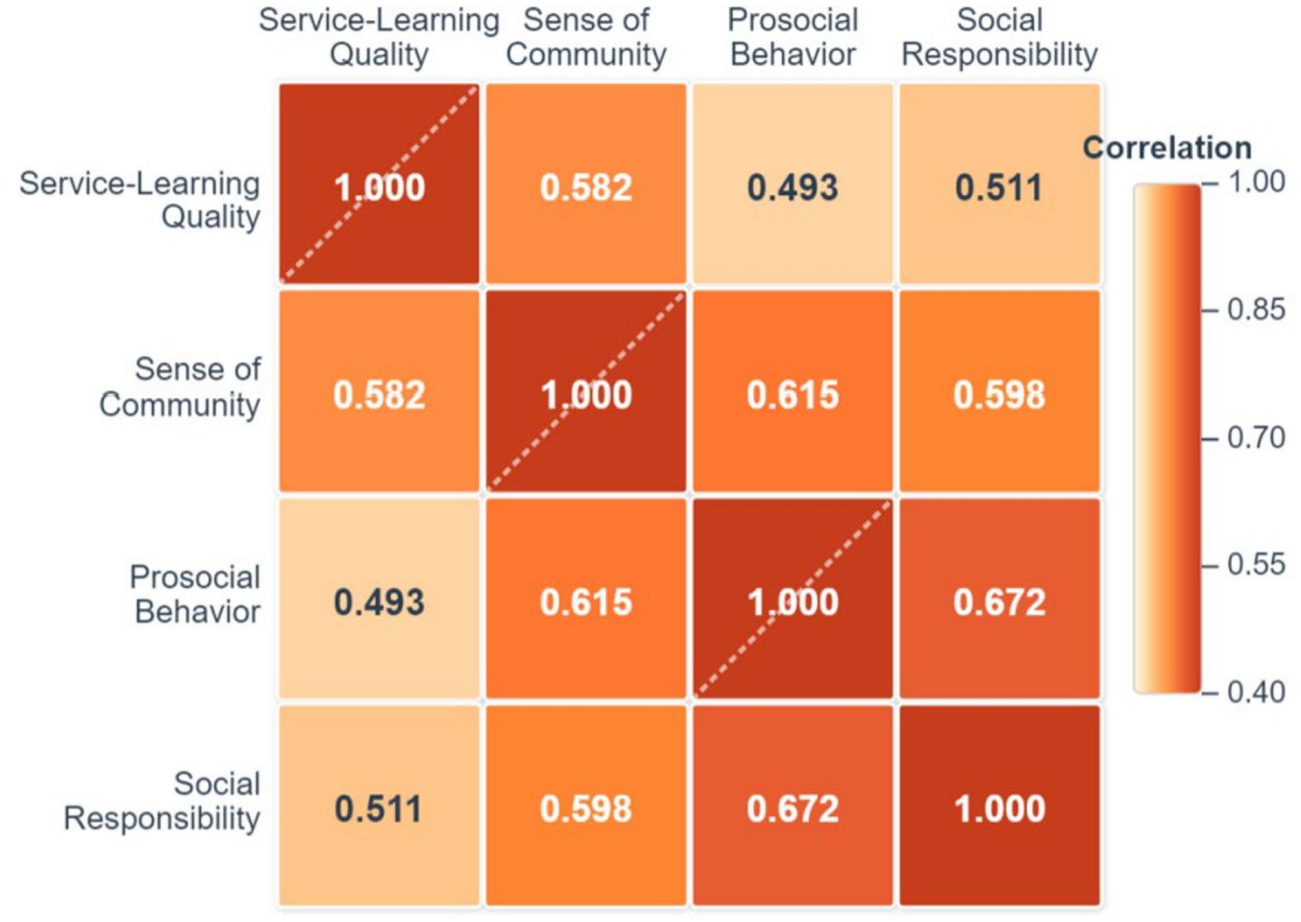
Heatmap of correlation coefficients among variables.

To explore the relationships between the variables, the present research used Pearson correlation analysis to calculate the correlation coefficients among the four main variables: service-learning quality, sense of community, prosocial behavior, and social responsibility. The correlation coefficient matrix is shown in Figure 2.

As shown in Figure 2, all main variables showed significant positive correlations (*p*-values for all correlations were less than 0.01). Specifically:

Service-learning quality was moderately and positively correlated with sense of community (*r = 0.582*), indicating that high-quality service-learning experiences are associated with the formation of a sense of team belonging among students.

Sense of community was moderately and positively correlated with both prosocial behavior (*r = 0.615*) and social responsibility (*r = 0.598*), suggesting that a sense of community is a key variable connecting the individual with social development.

Service-learning quality was also positively correlated with the two outcome variables (with prosocial behavior *r = 0.493*, and with social responsibility *r = 0.511*).

The correlation coefficients among the variables did not exceed 0.8, indicating that there were no serious multicollinearity issues.

These results provide preliminary validation for the study’s hypotheses that service-learning quality, sense of community, and students’ social development are closely related, laying the foundation for the subsequent mediation effect analysis.

### 4.5 Dimensional analysis of service-learning quality

To further investigate which specific component of service-learning quality contributes most significantly to student outcomes, a multiple regression analysis was conducted. Grounded in the study’s theoretical framework, “service-learning quality” was disaggregated into its three core sub-dimensions: Meaningful Service, Curriculum Integration, and Structured Reflection.

Two separate regression models were constructed, with Social Responsibility and Prosocial Behavior as the respective outcome variables. In each model, the three sub-dimensions were entered simultaneously as predictors. By comparing their standardized beta coefficients (*β*), it is possible to assess the relative importance of each dimension after controlling for the effects of the others. The results are detailed in Table 8.

**Table 8:** Multiple regression analysis of service-learning quality sub-dimensions on outcomes.

Outcome variable	Predictor sub-dimension	B	SE	β	t	*p*
Social responsibility	(Constant)	2.149	0.134		16.03	<0.001
	Meaningful service	0.141	0.035	0.150	4.07	<0.001
	Curriculum integration	0.153	0.030	0.186	5.08	<0.001
	Structured reflection	0.292	0.030	0.344	9.64	<0.001
	Model summary: R^2^ = 0.281, F(3, 731) = 95.35, *p* < 0.001					
Prosocial behavior	(Constant)	2.502	0.139		17.96	<0.001
	Meaningful service	0.170	0.036	0.175	4.73	<0.001
	Curriculum integration	0.052	0.031	0.061	1.66	0.097
	Structured reflection	0.237	0.031	0.271	7.59	<0.001
	Model summary: R^2^ = 0.158, F(3, 731) = 45.71, *p* < 0.001					

B = Unstandardized coefficient; SE = Standard Error; β = Standardized coefficient.

As shown in Table 8, the Structured Reflection dimension emerged as the most powerful predictor for both outcome variables. Specifically, it demonstrated the largest and most highly significant positive predictive effect on both Social Responsibility (*β = 0.344, p < 0.001*) and Prosocial Behavior (*β = 0.271, p < 0.001*). Although Meaningful Service and Curriculum Integration also had positive impacts, their influence was not as substantial as that of Structured Reflection. This finding underscores that within service-learning projects, guiding students to engage in purposeful and organized reflection is uniquely critical for promoting their personal and social development.

### 4.6 Mediation effect test

The SPSS process macro (model 4) was used to test the mediating role of sense of community in the association between service quality influencing social responsibility. Grade and gender variables were controlled. Bootstrap sampling was performed 5,000 times with a 95% confidence interval.

#### 4.6.1 Regression analysis results

The regression results for the mediation effect test are shown in Table 9. The data show that sense of community played a significant partial mediating role between service-learning quality and both outcome variables.

**Table 9:** Mediation effect test of sense of community on two outcome variables (*N* = 735).

Path/effect	Social responsibility		Prosocial behavior	
	β	95% CI	β	95% CI
Path coefficient				
a (service-learning quality → sense of community)	0.581***	0.510, 0.652	0.581***	0.510, 0.652
b (sense of community → outcome variable)	0.420***	0.361, 0.479	0.526***	0.467, 0.585
c’ (direct effect)	0.267***	0.202, 0.332	0.187***	0.122, 0.252
Effect size				
Total effect (c)	0.511***	0.457, 0.565	0.493***	0.432, 0.554
Indirect effect (a × b)	0.244***	0.189, 0.302	0.306***	0.252, 0.364
Mediation ratio	47.8%	—	62.1%	—
Model fit				
R^2^ (sense of community)	0.338	—	0.338	—
R^2^ (outcome variable)	0.421	—	0.465	—
*F*-value	267.31***	—	301.45***	—

***p < 0.001; 95% confidence intervals estimated using the Bootstrap method (5,000 resamples); control variables include gender and grade; path a is the same in both models because the mediator (sense of community) is the same.

#### 4.6.2 Mediation effect model

**Figure 3:**
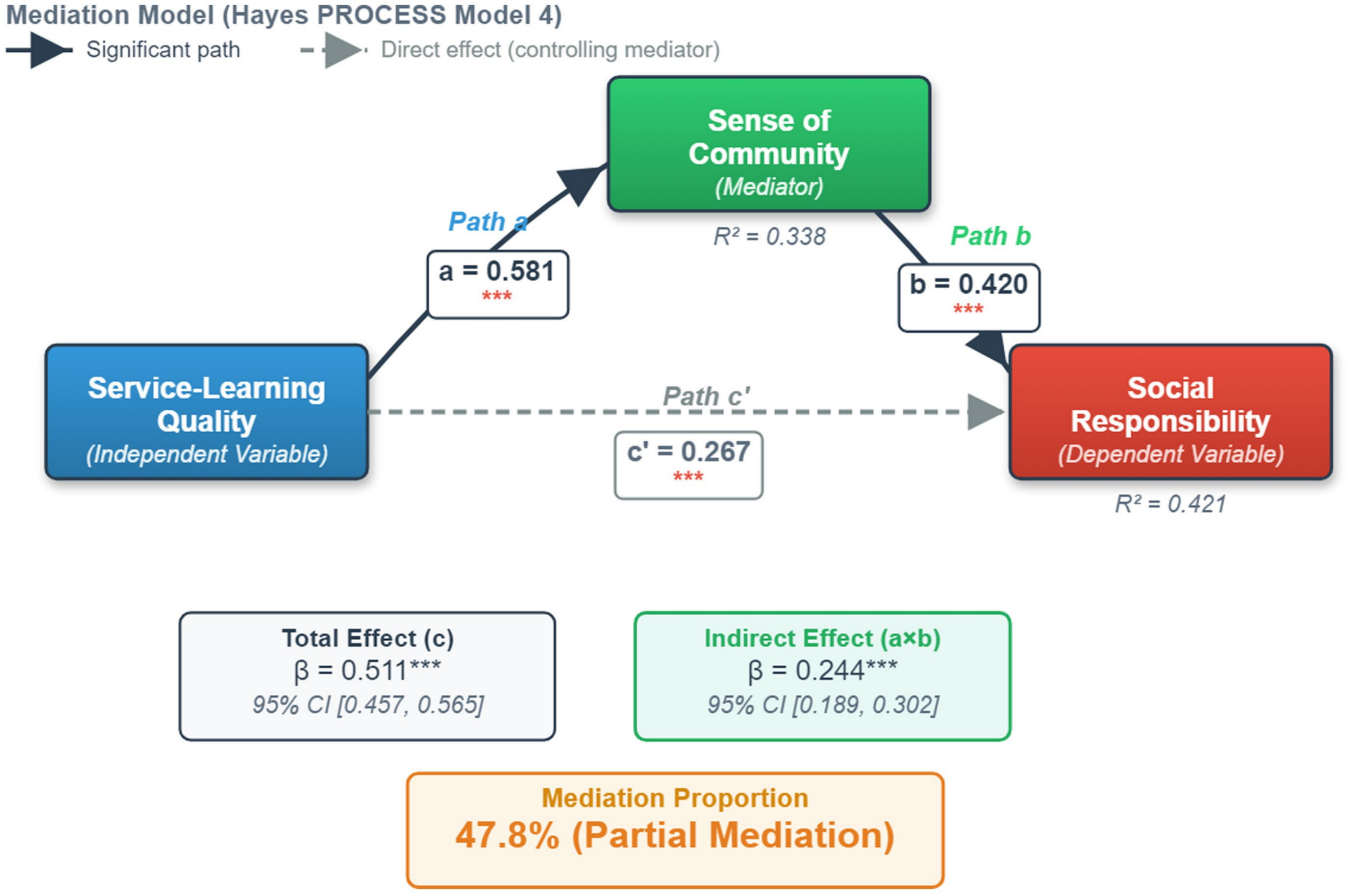
The mediation effect model of sense of community.

To more intuitively display the mediating role of sense of community between service-learning quality and social responsibility, the visualized model results are shown in Figure 3.

As shown in Figure 3, after controlling for gender and grade, the path analysis results further revealed the statistical associations between the variables:

Prediction path (path a): service-learning quality significantly and positively predicted sense of community (*β = 0.581*, *p < 0.001*). The variance explained by this path, R^2^ = 0.338, indicates that over one-third of the variation in sense of community can be explained by the organizational quality of service-learning, highlighting the importance of a high-quality practical environment for building a psychological community.
Mediation path (path b): after controlling for the influence of service quality, the positive predictive effect of sense of community on social responsibility remained significant (*β = 0.420*, *p < 0.001*), indicating that the emotional connection among students in the team is a core predictor of enhancing their sense of responsibility.
Mediation effect size: the overall explanatory power of the final model was R^2^ = 0.421, indicating that this mediation model can explain 42.1% of the variance in social responsibility. The Bootstrap test showed that the indirect effect value was 0.244 (95% CI [0.189, 0.302]), and the mediation contribution rate was 47.8%. This confirms that sense of community plays a crucial “bridge” role between “environmental input (service quality)” and “value output (social responsibility),” which is a typical partial mediation effect.

#### 4.6.3 Comparative analysis of mediation effects

To further explore whether the mediating role of sense of community differs for different outcome variables, the present research also examined the mediating effect of sense of community in the relationship between service-learning quality and prosocial behavior. As shown in Table 9, sense of community played a significant partial mediating role in both models, but there was a notable difference in the proportion of the mediation effect.

**Figure 4:**
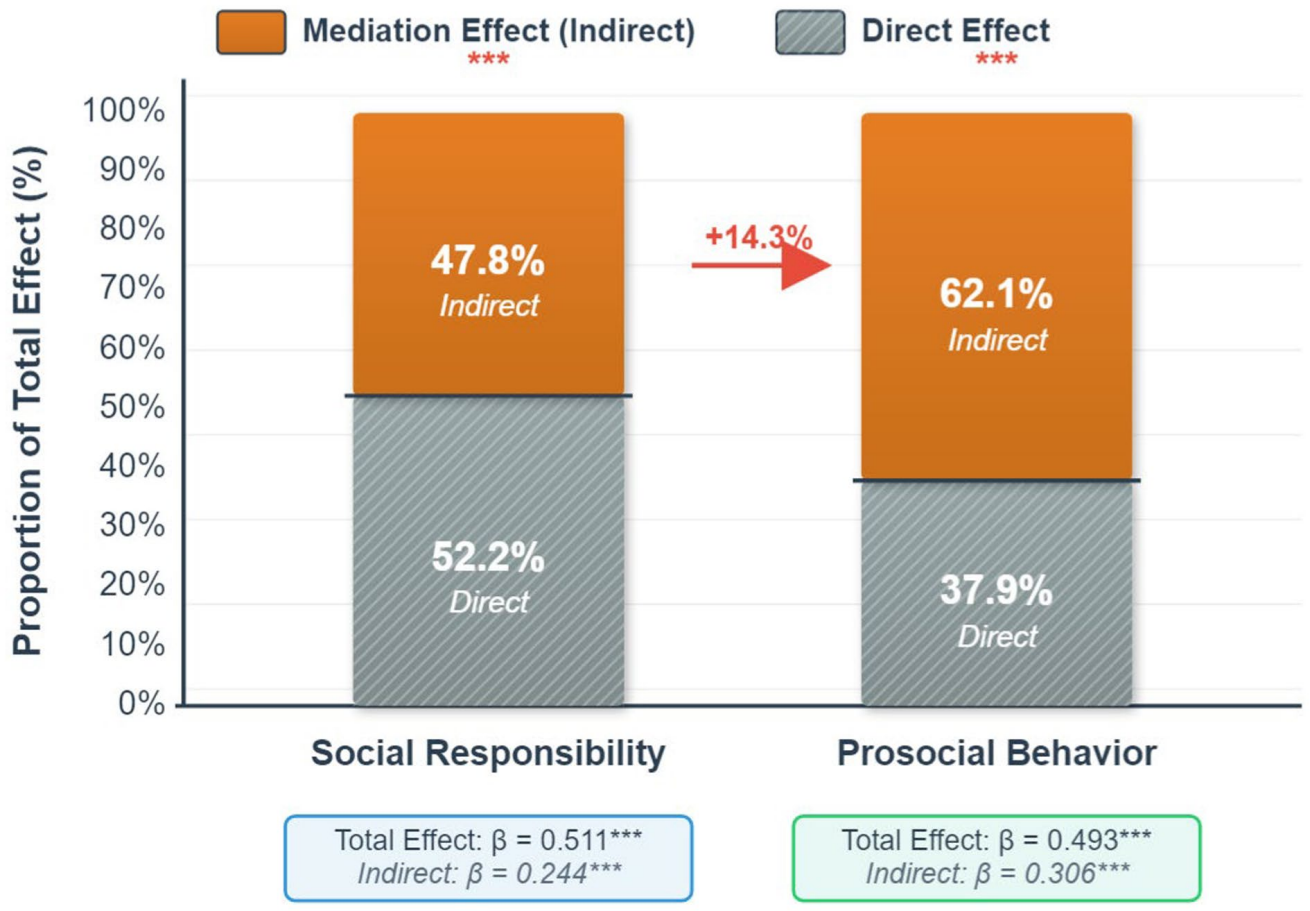
Comparison of the mediation effects of sense of community on different outcome variables.

For prosocial behavior, the indirect effect was *β* = 0.306 (95% CI [0.252, 0.364], *p < 0.001*), accounting for 62.1% of the total effect. For social responsibility, the indirect effect was β = 0.244 (95% CI [0.189, 0.302], *p < 0.001*), accounting for 47.8% of the total effect. This difference is visually presented in Figure 4.

As shown in Figure 4, the proportion of the mediation effect of sense of community on prosocial behavior (62.1%) was larger that on social responsibility (47.8%), a difference of 14.3 percentage points. This finding has important theoretical implications:

The results suggest that a sense of team belonging may have a stronger association with an individual’s tendency to engage in helping behaviors. This is consistent with the predictions of social identity theory (Tajfel et al., 2001)—when individuals gain a strong sense of belonging in a team, they are more likely to internalize the team’s values and exhibit altruistic behavior in their daily lives. In contrast, the association between a sense of community and social responsibility may require the combined action of other factors (such as personal values, moral cognition, etc.). To formally test this difference, we conducted a Bootstrap contrast analysis comparing the two indirect effects. The analysis revealed that the indirect effect on prosocial behavior (*β = 0.306*) was significantly stronger than the indirect effect on social responsibility (*β = 0.244*). The difference between these two effects was statistically significant (Effect Difference = 0.062, 95% Bootstrap CI [0.008, 0.121]), as the confidence interval does not contain zero. This statistically confirms that the mediating role of sense of community is more pronounced in its association with prosocial behavior.

From the perspective of path coefficients, the predictive strength of sense of community on prosocial behavior (*β = 0.526*) was stronger than its influence on social responsibility (*β = 0.420*), further suggesting the unique role of team atmosphere in promoting specific behaviors. Prosocial behavior, as an overt and observable behavior pattern, is more susceptible to immediate social contexts (such as team support, peer role models), while social responsibility, as an implicit attitudinal tendency, is formed under the comprehensive influence of multiple factors.

Although the mediation effect proportions differ, the direct effects in both models were significant (*p < 0.001*), indicating that service-learning quality, in addition to being associated with student development through a sense of community, may also have an effect through other pathways (such as skill enhancement, increased self-efficacy, etc.). This provides a direction for future research to explore multiple mediation models.

In summary, based on the results from Table 9 and Figure 4, it can be concluded that a sense of community plays a key mediating role in the educational process of service-learning, but its mode of action varies depending on the nature of the outcome variable. For behavioral development (prosocial behavior), the role of team belonging is more direct and significant; whereas for attitudinal development (social responsibility), more influencing factors need to be considered.

## 5 Discussion

The present research aimed to explore how service-learning is associated with the social development of university students through psychological mechanisms. Adopting a psychosocial perspective we moved beyond this educational process through a simple “input–output” model, but instead focus on how emotional experience (sense of community) acts as a core mediating factor linking service-learning to student outcomes.

### 5.1 High-quality service and deep engagement: a potential starting point to counter “academic alienation”

The findings show that the quality of service-learning significantly and positively predicts social responsibility (*β = 0.511*) and prosocial behavior (*β = 0.493*). This result reiterates Dewey’s view that education is life. When learning moves out of the classroom and connects with real social needs, it gains the power to potentially counter feelings of “academic alienation.” This is consistent with recent findings by Anderson et al. (2025) and Alfirević et al. (2023), who pointed out that participation in civic activities and addressing real challenges are effective ways to alleviate youth anxiety and rebuild social connections. Notably, our finding that “structured reflection” was the most significant correlate of strongest influence (*β = 0.344, p < 0.001*) aligns perfectly with Kolb’s experiential learning theory, which posits that the depth of learning is closely linked to the process of “meaning-making.” High-quality reflection appears to transform scattered service experiences into a systematic cognitive structure, allowing students to realize that what they learn is not just for exams, but for solving real-world problems (Compare and Albanesi, 2024). This sense of self-efficacy is the cognitive foundation for students to regain their learning Engagement (Gutzweiler et al., 2022).

### 5.2 Sense of community: “meaning internalization” triggered by emotional resonance

The study’s central finding is the identification of sense of community as a critical psychological mediator. This single factor accounted for a substantial portion of the outcomes: 47.8% in the social responsibility model and 62.1% in the prosocial behavior model. The magnitude of these effects indicates that a significant part of the educational impact is statistically mediated through emotional connection. This outcome suggests that the “meaning” of learning, such as developing social responsibility, is associated less with external moral preaching and more from genuine emotional resonance (Russo et al., 2025). Self-Determination Theory (SDT) provides a theoretical basis for this, positing that fulfilling the innate ‘need for relatedness’ is a fundamental driver of intrinsic motivation. When students in a service team feel “accepted” and that “everyone is important” (sense of community), this sense of belonging not only brings a pleasant experience (joy) but also provides a psychological Secure Base (Li et al., 2025). Within this sense of security, students dare to explore the external world, spontaneously generate prosocial behavior (62.1% mediation ratio), and this experience is associated with the internalization of long-term social responsibility (Marino et al., 2025). Furthermore, small teams (2–10 members) have a significantly higher sense of community than large teams. The psychological mechanism behind this may be that small-scale teams provide more frequent and deeper “the positive learning experiences” (Henderson, 2025). In a moderately sized group, an individual’s voice is more likely to be heard, and this return of subjectivity may inhibit “social loafing” and is the basis for achieving true “positive engagement”.

Service duration plays a significant role in shaping students’ outcomes. Long-term engagement (≥8 days) was associated with stronger sense of community, prosocial behavior, and social responsibility compared to short-term projects. This aligns with contact theory (Pettigrew and Tropp, 2006; Tajfel et al., 2001), which posits that sustained intergroup contact facilitates deeper understanding and emotional bonds. The temporal dimension suggests that meaningful internalization requires not only emotional resonance but also sufficient time for reflection and relationship-building. Short-term “hit-and-run” service may generate temporary enthusiasm, but lacks the depth needed for lasting value transformation.

### 5.3 Practical implications: shifting from “task completion” to “meaningful engagement”

Based on the above findings, to help students regain engagement and meaning through service-learning in higher education, we propose the following suggestions directly grounded in the study’s results:

First, based on the finding that sense of community is a key mediator, educators should aim to create an “emotional community” to awaken intrinsic engagement. Ideological and political education and practical teaching should shift their focus from “what was done” to “what was felt.” Universities should strive to build service teams into emotional communities through icebreakers, team-building activities, and other means. Only when students first feel happy and accepted in the group (Joy) can they truly open their hearts to embrace a broader sense of social responsibility (Guanlao et al., 2025; Ma et al., 2025).

Second, our results showing that small teams report a significantly higher sense of community support the promotion of a “micro-team” model to ensure interaction quality. Additionally, given that long-term engagement (≥8 days) further enhances these outcomes, universities should prioritize sustained service projects over one-time events. Given the high sense of community in small teams, it is recommended to implement a “project-based small group system” (5–8 members) in large-scale practical activities. Close interaction in small groups can maximize each student’s sense of participation, avoid the “free-rider” phenomenon, and allow every student to experience “my importance to others” (Vázquez et al., 2025).

Third, given the strong predictive power of “structured reflection” on outcomes, it is crucial to deepen narrative reflection to promote meaning internalization. Teachers should guide students to engage in “narrative reflection.” Existing research shows that active guidance and participation from teachers (teaching presence) can significantly enhance students’ behavioral engagement (Wang, 2024). Therefore, instead of just recording service hours, students should be encouraged to tell moving stories and share emotional moments from their service. This helps them reaffirm the value of their actions in retrospect, thus completing the psychological loop from “emotional experience” to “value establishment” (De Oliveira, 2024).

### 5.4 Limitations and future prospects

Although the present research reveals important psychological associations, it still has limitations:

First, the cross-sectional design limits causal inference. Although the model was built based on theory, it cannot establish temporal precedence or causality. The associations found are correlational. Future research is advised to adopt a Longitudinal Design to capture the trajectory of students’ psychological changes before, during, and after service, which would allow for a stronger inference of causality (Ma et al., 2025).

Second, the study’s reliance on self-report data presents a potential for common method bias (CMB). While procedural safeguards were implemented and subsequent statistical checks (including a full collinearity VIF test) suggested that CMB did not significantly distort the findings, the single-source nature of the data remains a methodological limitation. It is worth noting, however, that subjective experiences like “sense of community” are often best captured directly through self-report. To build upon our findings, future research could strengthen the validity through triangulation, incorporating multi-source data such as peer ratings or teacher observations to more comprehensively assess students’ behavioral performance (Kor et al., 2025).

Third, the mediation models did not include all contextual factors as covariates. Our preliminary analyses in Section 4.3 confirmed that team size and service type significantly influence the sense of community and other outcomes. However, to maintain the parsimony of the core theoretical model and to clearly present the primary mediation pathway, these contextual factors were analyzed separately rather than as covariates in the main mediation analysis. This is a limitation, as these factors could potentially confound the mediation effects. Future research should consider employing more complex models, such as a moderated mediation model, to simultaneously test how these contextual variables alter the strength of the mediation pathways.

Fourth, the study focused exclusively on the positive outcomes of service-learning and sense of community. While our findings highlight the benefits, the literature also suggests that high levels of engagement and responsibility can sometimes lead to stress, emotional exhaustion, or burnout, particularly in person-centered service roles. Our model did not include measures to capture these potential negative effects. Future studies should adopt a more balanced perspective by simultaneously examining both the positive and negative psychological consequences of service-learning to provide a more holistic understanding.

Finally, the sample was mainly from universities in eastern China. Considering the special emphasis on “relationships” in Chinese collectivist culture, the mediating effect of sense of community might be overestimated. Future research could conduct cross-cultural comparisons to explore the universality of this mechanism in different cultural backgrounds (Camus et al., 2022).

## 6 Conclusion

The present research constructed and validated a statistical model of the associations among Service-Learning Quality, Sense of Community—University Students’ Social Development. The results indicate that service-learning is not only a learning field for knowledge application but also a key pathway for students to reconstruct the Meaning of learning through social connection.

The findings confirm that sense of community, as a core mediating variable, explains nearly half of the effect of service-learning on students’ social responsibility and prosocial behavior. This suggests that emotional resonance and a sense of belonging are the important factors associated with students to shift from “passively completing tasks” to “actively taking responsibility.” Furthermore, small-scale teams and direct, person-centered service are more effective in fostering students’ intrinsic engagement.

In conclusion, educators should not overlook the emotional dimension of service-learning. By building a warm and nurturing educational community, we can provide opportunities for students to experience positive emotions in “helping others” and establish “responsibility” in “connection,” thereby contributing to the educational goal of helping students find meaning and curiosity in their learning journey.

## Data Availability

The original contributions presented in the study are included in the article/Supplementary material, further inquiries can be directed to the corresponding author.
