# Supplementary material for: Building a sense of community in service-learning and its association with college students’ prosocial behavior and social responsibility
Source: Front Psychol. 2026 Mar 24;17:1796279. doi: 10.3389/fpsyg.2026.1796279 (PMC13055623; doi:10.3389/fpsyg.2026.1796279)
Supplement: Supplementary file 1 [file Data_Sheet_1.ZIP › Data Coding Book.docx]

# **Data Coding Book**

## **Description**

This file describes the variables contained in the dataset cleaned_data_735.csv. It serves as a guide for researchers to understand the variable names, measurement scales, and value codes used in the statistical analysis.

## **1. Demographic and Contextual Variables**

| Variable | Label | Value Codes (Coding Scheme) |
| --- | --- | --- |
| Q1_Gender | Gender | 1 = Male, 2 = Female |
| Q2_Grade | Academic Year | 1 = Freshman, 2 = Sophomore, 3 = Junior, 4 = Senior, 5 = Graduate Student |
| Q3_Major | Field of Study | 1 = Humanities/Social Sciences, 2 = Science/Eng/Agri/Med, 3 = Arts/Sports, 4 = Other |
| Q4_Participation | Participation Check | 1 = Yes (Dataset strictly includes ‘Yes’ only) |
| Q5_Duration | Service Duration | 1 = < 1 day, 2 = 2-7 days, 3 = 8-30 days, 4 = > 1 month |
| Q6_TeamSize | Team Size | 1 = 2-5 members, 2 = 6-10 members, 3 = 11-20 members, 4 = > 20 members |
| Q7_Target | Service Recipient | 1 = Children, 2 = Elderly, 3 = Disabled, 4 = Residents, 5 = Rural Revitalization, 6 = Environment, 7 = Other |

## **2. Measurement Scales (Likert Scale Variables)**

*Note:*

**SLQ** = Service-Learning Quality (Independent Variable)

**SOC** = Sense of Community (Mediator)

**PSB** = Prosocial Behavior (Dependent Variable 1)

**SR** = Social Responsibility (Dependent Variable 2)

*Scale:* 1 = Strongly Disagree (or Never) to 5 = Strongly Agree (or Always).

| Variable | Item Key Word / Short Description |
| --- | --- |
| Q8_SLQ | Service was urgently needed |
| Q9_SLQ | Positive impact on others |
| Q10_SLQ | Applied professional knowledge |
| Q11_SLQ | Deeper understanding of theory |
| Q12_SLQ | Organized reflection time |
| Q13_SLQ | Reflected on values/career |
|  |  |
| Q14_SOC | Membership: True member |
| Q15_SOC | Membership: Sense of belonging |
| Q16_SOC | Influence: Opinions taken seriously |
| Q17_SOC | Influence: Influence on direction |
| Q18_SOC | Needs: Learned things |
| Q19_SOC | Needs: Support from teammates |
| Q20_SOC | Connection: Shared goals/values |
| Q21_SOC | Connection: Shared impressive moments |
|  |  |
| Q22_PSB | Share opportunities |
| Q23_PSB | Help friends in difficulty |
| Q24_PSB | Comfort people feeling down |
| Q25_PSB | Benefit the group |
| Q26_PSB | Empathize with misfortunes |
| Q27_PSB | Help strangers (e.g., lost passerby) |
|  |  |
| Q28_SR | Contribute to overall society |
| Q29_SR | Moral obligation to help |
| Q30_SR | Participate in public affairs |
| Q31_SR | Persist in promises |
| Q32_SR | Act against injustice |
| Q33_SR | Consider consequences on society |
