# Supplementary material for: Building a sense of community in service-learning and its association with college students’ prosocial behavior and social responsibility
Source: Front Psychol. 2026 Mar 24;17:1796279. doi: 10.3389/fpsyg.2026.1796279 (PMC13055623; doi:10.3389/fpsyg.2026.1796279)
Supplement: Supplementary file 1 [file Data_Sheet_1.ZIP › Appendix A Survey Questionnaire.docx]

Supplementary Material 1: Survey Questionnaire

Paper Title: Building a Sense of Community in Service-Learning and Its Impact on College Students' Prosocial Behavior and Social Responsibility

Description: This document presents the original survey instrument used for data collection in the study. The questionnaire was distributed in Chinese and translated into English for this submission.

Dear Student,

Hello! This is an academic survey questionnaire about university students’ service-learning experiences and personal growth. This research aims to understand your feelings and takeaways from participating in social practice, volunteer services, and similar activities, with the hope of providing a scientific reference for better organizing such activities in the future.

This survey will be conducted completely anonymously. All data will be used for aggregate statistical analysis only. We promise to keep your personal information and responses strictly confidential, so please feel free to answer honestly. Completing the questionnaire will take approximately 5-10 minutes. Your truthful answers are crucial to this research!

You may choose to stop answering at any time during the process. By completing and submitting the entire questionnaire, you indicate that you are informed and consent to participate in this survey.

Thank you very much for your support and cooperation!

Part I: Basic Information and Service-Learning Participation

**Instructions:** The following questions are designed to understand your basic information and background regarding your participation in service-learning. Please fill in or select according to your actual situation.

**1. Your gender:**
○ Male ○ Female

**2. Your academic year:**
○ Freshman ○ Sophomore ○ Junior ○ Senior ○ Graduate Student

**3. Your field of study:**
○ Humanities & Social Sciences ○ Science, Engineering, Agriculture & Medicine ○ Arts & Sports ○ Other

**4. In the past year, have you participated in any social practice or volunteer service activities with an off-campus community service component, organized by your university, college, or a student club?**
○ Yes ○ No (Thank you for your participation. This is the end of the questionnaire.)

**5. [Key Context] Please recall your most memorable service-learning experience and answer all subsequent questions based on this experience. The total duration of this project was approximately:**
○ Within 1 day ○ 2-7 days ○ 8-30 days ○ More than 1 month

**6. In this project, the size of your team was approximately:**
○ 2-5 people ○ 6-10 people ○ 11-20 people ○ More than 20 people

**7. The primary service recipients of this project were:**
○ Children/Adolescents ○ The Elderly ○ Persons with Disabilities ○ Community Residents ○ Related to rural revitalization ○ Related to environmental protection ○ Other __________

Part II: Service-Learning Quality

**Instructions:** Please recall the activity you mentioned above and evaluate the actual situation. (1=Strongly Disagree, 5=Strongly Agree)

| Item | 1 Strongly Disagree | 2 Disagree | 3 Neutral | 4 Agree | 5 Strongly Agree |
| --- | --- | --- | --- | --- | --- |
| 8. The service I provided was genuinely and urgently needed by the community or the recipients. | ○ | ○ | ○ | ○ | ○ |
| 9. I could clearly feel that my work had a positive impact on others. | ○ | ○ | ○ | ○ | ○ |
| 10. I applied professional knowledge or skills learned in class during the activity. | ○ | ○ | ○ | ○ | ○ |
| 11. This service experience gave me a deeper understanding of theoretical knowledge that I had found dry. | ○ | ○ | ○ | ○ | ○ |
| 12. The teacher or organizer arranged specific times (e.g., summary meetings) for us to share our feelings and reflect. | ○ | ○ | ○ | ○ | ○ |
| 13. I often reflected on the impact of this experience on my personal values or future career plans. | ○ | ○ | ○ | ○ | ○ |

Part III: Sense of Community

**Instructions:** Please rate your true feelings about the “service team/group” you worked with at the time. (1=Strongly Disagree, 5=Strongly Agree)

| Item | 1 Strongly Disagree | 2 Disagree | 3 Neutral | 4 Agree | 5 Strongly Agree |
| --- | --- | --- | --- | --- | --- |
| 14. (Membership) I felt like a true member of this team, not just a passerby. | ○ | ○ | ○ | ○ | ○ |
| 15. (Membership) I had a strong sense of belonging in this team; everyone accepted me. | ○ | ○ | ○ | ○ | ○ |
| 16. (Influence) When making decisions or solving problems, my opinions were taken seriously. | ○ | ○ | ○ | ○ | ○ |
| 17. (Influence) I felt I could have a certain degree of influence on the direction of the team’s work. | ○ | ○ | ○ | ○ | ○ |
| 18. (Needs Fulfillment) Participating in this team helped me learn things; it wasn’t a waste of time. | ○ | ○ | ○ | ○ | ○ |
| 19. (Needs Fulfillment) When the team encountered difficulties, I could get support and help from my teammates. | ○ | ○ | ○ | ○ | ○ |
| 20. (Emotional Connection) I feel that the team members shared common goals and similar values. | ○ | ○ | ○ | ○ | ○ |
| 21. (Emotional Connection) The moments (of challenge or success) we experienced together remain impressive to me. | ○ | ○ | ○ | ○ | ○ |

Part IV: Prosocial Behavior Tendency

**Instructions:** Please rate the likelihood of you exhibiting the following behaviors in your daily life. (1=Never, 5=Always)

| Item | 1 Never | 2 Rarely | 3 Sometimes | 4 Often | 5 Always |
| --- | --- | --- | --- | --- | --- |
| 22. I willingly share opportunities I have (e.g., job information, learning resources) with others. | ○ | ○ | ○ | ○ | ○ |
| 23. I am willing to spend time helping friends or classmates in difficulty, without expecting anything in return. | ○ | ○ | ○ | ○ | ○ |
| 24. I try to comfort people who are sad, depressed, or feeling down. | ○ | ○ | ○ | ○ | ○ |
| 25. Even if it doesn’t directly benefit me, I will do things that are good for my class or the group. | ○ | ○ | ○ | ○ | ○ |
| 26. I easily empathize and feel compassion for the misfortunes of others. | ○ | ○ | ○ | ○ | ○ |
| 27. When I see someone who needs help (e.g., a lost passerby), I will lend a hand without hesitation. | ○ | ○ | ○ | ○ | ○ |

Part V: Social Responsibility

**Instructions:** Please rate your level of agreement with the following statements. (1=Strongly Disagree, 5=Strongly Agree)

| Item | 1 Strongly Disagree | 2 Disagree | 3 Neutral | 4 Agree | 5 Strongly Agree |
| --- | --- | --- | --- | --- | --- |
| 28. Everyone should contribute to the overall benefit of society, not just care about their own gains and losses. | ○ | ○ | ○ | ○ | ○ |
| 29. Even if not legally required, I feel a moral obligation to help those less fortunate than me. | ○ | ○ | ○ | ○ | ○ |
| 30. Participating in public affairs (e.g., community governance, public service activities) is a duty for every qualified citizen. | ○ | ○ | ○ | ○ | ○ |
| 31. If I promise to do something, I will persist in completing it, even if it becomes very difficult later on. | ○ | ○ | ○ | ○ | ○ |
| 32. When I see injustice in society, I feel uneasy and hope to do something to change it. | ○ | ○ | ○ | ○ | ○ |
| 33. When making decisions, I often consider the consequences of my actions on the people around me and on society. | ○ | ○ | ○ | ○ | ○ |

This is the end of the questionnaire. Thank you again for your sincere help! Wishing you success in your studies and a happy life!
